# Consumption of Phenolic-Rich Food and Dietary Supplements as a Key Tool in SARS-CoV-19 Infection

**DOI:** 10.3390/foods10092084

**Published:** 2021-09-03

**Authors:** José David Flores-Félix, Ana C. Gonçalves, Gilberto Alves, Luís R. Silva

**Affiliations:** 1 CICS-UBI–Health Sciences Research Centre, Faculty of Health Science, University of Beira Interior, 6200-506 Covilhã, Portugal; jdflores@usal.es (J.D.F.-F.); anacarolinagoncalves@sapo.pt (A.C.G.); gilberto@fcsaude.ubi.pt (G.A.); 2 Unidade de Investigação para o Desenvolvimento do Interior (UDI/IPG), Instituto Politécnico da Guarda, 6300-559 Guarda, Portugal

**Keywords:** dietary supplements, COVID-19, immune response, mediterranean diet, phenolic compounds

## Abstract

The first cases of COVID-19, which is caused by the SARS-CoV-2, were reported in December 2019. The vertiginous worldwide expansion of SARS-CoV-2 caused the collapse of health systems in several countries due to the high severity of the COVID-19. In addition to the vaccines, the search for active compounds capable of preventing and/or fighting the infection has been the main direction of research. Since the beginning of this pandemic, some evidence has highlighted the importance of a phenolic-rich diet as a strategy to reduce the progression of this disease, including the severity of the symptoms. Some of these compounds (e.g., curcumin, gallic acid or quercetin) already showed capacity to limit the infection of viruses by inhibiting entry into the cell through its binding to protein Spike, regulating the expression of angiotensin-converting enzyme 2, disrupting the replication in cells by inhibition of viral proteases, and/or suppressing and modulating the host’s immune response. Therefore, this review intends to discuss the most recent findings on the potential of phenolics to prevent SARS-CoV-2.

## 1. Introduction

The current pandemic coronavirus disease-2019 (COVID-19) is probably the most convulsive global event in the history of mankind and it is caused by the severe acute respiratory syndrome coronavirus 2 (SARS-CoV-2). Its emergency occurred at the end of the year 2019 in China, where the alarms about transmissibility and morbidity were lit, because on January 19 of 2020, of the 189 people with COVID-19, only 19 did not require hospitalization, and three were detected in other countries without any relation to the possible focus, which was reported as a local food market in China [1]. 

Different taxonomic studies have shown that this virus is a member of the so-called SARS-related coronaviruses (SARSr-CoV) and belongs to the Coronaviridae family and *Betacoronavirus* genus. Although this one is considered a zoonosis, the final vector has not been detected yet. However, the genomic analysis shows important similarities with other Betacoronaviruses isolated from bats, such as *Rhinolophus affinis*, or other mammals as civets and pangolins. Therefore, the most widespread hypothesis is that horizontal gene transfer and recombination events occurred involving bat RaTG13 and Guangdong pangolin coronaviruses, and bat CoV ZC45 and ZXC21 strains [2,3,4,5].

**Figure 1:**
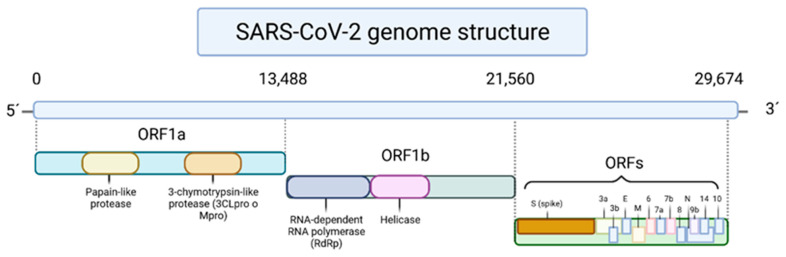
Genomic scheme of SARS-CoV-2 genome.

In a general way, the SARS-CoV-2 is composed of open reading frames, where Open Reading Frame 1a and 1b (ORF1a and ORF1b, respectively) are responsible to encode polyproteins of the SARS-CoV-2 genome at the 5′ end (Figure 1), including the non-structural 3-chymotrypsin-like protease (3CL^pro^ or M^pro^), papain-like protease (PL^pro^) and RNA-dependent RNA polymerase (RdRp). In the other side of the virus chain, i.e., at the terminal 3′, there are found ORFs that encode the viral surface proteins, namely the Spike (S), envelope (E), membrane (M) and nucleocapsid (N) proteins [6]. Like other SARSr-CoVs, SARS-CoV-2 also uses the highly glycosylated S protein located on its viral membrane to bind the host cell receptor, the angiotensin-converting enzyme II (ACE2), and thus, enter into the human body [7]. This enzyme is present in the membrane of many cells of different organs and plays a crucial role in the regulation of blood pressure and electrolyte balance [8]. The linkage of the S protein generates an inactivation of the ACE2 protein, causing an imbalance in the proportion of peptides generated by angiotensin-converting enzyme I (ACE1) and ACE2, and triggering a pre-inflammatory response, which causes, in most cases, symptoms similar to flu [8,9]. Additionally, transmembrane protease serine 2, which is an enzyme encoded by the TMPRSS2 gene in humans, is also involved in the cleavage of S protein and ACE2, allowing the viral endocytose [10]. The infection with this virus promotes the appearance of fever, dry cough, fatigue, dyspnea, myalgias, headache, sore throat, rhinorrhea, and gastrointestinal symptoms [6]. Sometimes, the exaggerated and uncontrolled immune system response, compromise vital functions and damage organs, leading to the development of pneumonia and eventual death, independently of age, gender, and health condition. Furthermore, a characteristic that makes this disease remarkable is the appearance of persistent symptoms. Indeed, statistical data indicates that around 50% of the infected do not return to their initial health state, presenting continuous fatigue and higher levels of C-reactive protein and lactate dehydrogenase, which can be synonyms with cell death [9].

Normally, the COVID-19 is treated with paracetamol. However, it is well-known that the continuous intake of pharmaceutical drugs is not recommended due to their undesirable side effects. Additionally, and since it is also recognized that the rapid spread of the virus throughout the world collapsed most health systems and decreased economic activities of the countries, several efforts have been done to attenuate, or even mitigate, this pandemic disease [11,12]. In this order of ideas, it is not surprising that other therapeutical strategies are being studied and developed, including those based on phenolics. In fact, phenolics present several advantages, as low toxicity, predominance in nature, and abilities to relieve oxidative stress, reduce inflammatory markers and restore the immune system. Particularly, some cellular studies and docking assays already showed that these compounds can interact with the virus structure, avoiding their entrance to cells and consequent replication. So, the main goal of the present review is to assess and elucidate the most recent findings regarding the potential of phenolics to prevent SARS-CoV-2.

## 2. Phenolic Compounds in Human Health

Phenolic compounds are a heterogeneous group of molecules whose central structure can be only a hydroxybenzene or phenol ring (monophenols, e.g., gallic acid, p-coumaric), or composed of two (e.g., stilbenes, dimeric acids), three (e.g., quercetin, genistein), or more joined rings (e.g., proanthocyanins, tannins), the so-called polyphenols. Up to date, more than 50,000 different phenolic compounds are known, and they are classified according to their central structure and radical substituents, being differentiated between two main groups, which are, non-flavonoids and flavonoids [13]. Non-flavonoids compounds are composed of phenolic acids such as hydroxybenzoic and hydroxycinnamic acids, lignans, coumarins and stilbenes, highlighting among them gallic acid, caffeic acid, p-coumaric acid and resveratrol. On the other hand, flavonoids are the most abundant phenolic compounds in plants and, contrary to the previous ones, they are characterized by being a broader set of molecules, being subdivided into flavonols, flavan-3-ols, flavones, flavanones, isoflavones, flavonols and anthocyanins. Among them, kaempferol, catechin, epicatechin, apigenin, hesperetin, naringenin, genistein, cyanidin and delphinidin are the predominant ones [14,15,16]. All of them have in common the characteristic of being synthesized as plant secondary metabolites where they fulfill a wide range of biological functions. Phenolics can confer protection against ionizing radiation, respond to biological aggressions by secreting phytoalexins, act as antibacterial and antifungal agents, as well as attractants for pollinators, in addition to having capacity to accumulate certain molecules capable to modify the coloration of different organs [17].

Like humans and other animals (Metazoa) are not able to synthetize phenolics, their obtainment comes from the uptake of fruits, vegetables, medicinal plants, food supplements, among others. Their consumption is extremely important and beneficial given their notable antioxidant and anti-inflammatory properties [18]. However, their acquisition is limited owing to their bioavailability, which depends on multiple factors, such as the molecule itself, the intestinal microbiota, pH values and the consumption with other compounds. Furthermore, it is also important to take into account the inter-variability between individuals. All of these factors contribute to the different kinetic characteristics shown by each compound [19]. Currently, the polyphenols with the highest bioavailability are phenolic acids, followed by isoflavones, flavonols, catechins, flavanones, proanthocyanidins and lastly anthocyanins. However, recent studies revealed that, probably, flavanones and anthocyanins can be more bioavailable than previously reported once they suffer an extensive metabolization in intestinal microbiota [20,21]. Particularly, and focusing on anthocyanins, Ludwig et al. [22] and Mueller et al. [23] reported that cyanidin glycosides, after metabolization, can originate around 35 different metabolites, where the main ones are 2,4,6-trihydroxybenzaldehyde, p-coumaric, protocatechuic and vanillic acids, and phenolic conjugates (e.g., hippuric, phenylacetic, and phenylpropenoic acids).

Over the last few decades, the vision about phenolic compounds has changed drastically. If before they were considered xenobiotics with the ability to reduce the absorption of proteins and other bioactive compounds, today, their presence in food is increasingly important, as many depth studies have shown their ability to counteract oxidative stress, and therefore, preventing, or attenuating the symptoms of many chronic diseases, such as diabetes and cardiovascular pathologies, and also to control weight [24,25]. These compounds already showed ability to regulate the appetite and lipid metabolism, inhibit the differentiation of adipocytes and serve as beneficial gut microbiota prebiotics [26]. Furthermore, they are able to reduce the activity of disaccharidases (i.e., *α*-glucosidase and *α*-amylase) and the absorption of sugars, and improve the use of monosaccharides by muscle cells [27]. These capacities are essentially due to their chemical, standing out the presence of multiple hydroxyl groups, which can easily interact with gastrointestinal enzymes involved in carbohydrate metabolism, and hence, interfering with their functions [28]. In the same way, other enzymatic activities have been described regarding phenolics, such as the ability to inhibit the DNA polymerases α and δ (which are involved in cells proliferation), as well as to interact with zinc metalloproteinases, including those involved in ACE system [29]. Lastly, their consumption also shows to have a positive effect on the incidence of cardiovascular diseases, observing a direct relationship between the consumption of these compounds and the reduction of the risk of hypertension, dyslipidemia, coronary and arterial diseases events [30,31]. In this way, phenolic compound consumption has been related to a vasodilator effect at the peripheral level, such as in the endothelium, relating this effect to the management of oxidative stress and the blocking of reactive oxygen species [19,32,33]. Specifically, quercetin and resveratrol, have been shown to be efficient inhibitors of the signaling pathway of the protein Mammalian Target of Rapamycin, which is related to problems of arteriosclerosis, cardiac muscle degeneration and vascular integrity [34]. Besides, both compounds can also regulate the concentration of low-density lipoprotein cholesterol in the blood, improve the oxidative balance due to their high antioxidant capacities and reduce the degenerative effects associated with this metabolic state [31]. In addition, resveratrol, cherries’ cinnamic acids and anthocyanins have been shown capacity to regulate the basal levels of the control systems of circadian rhythms, through the modulation of the expression of CLOCK-BMAL1 genes. Since these genes are involved in the determination of liver sensitivity to insulin and are affected by dark cycles, the action of anthocyanins will allow restoring the correct metabolization of fatty acids [35].

Besides, phenolics also possess antimicrobial activities. They already showed capacity to interfere with the growth of *Escherichia coli* H157: H7, *Salmonella* sp., *Listeria monocytogenes* and *Citrobacter*, with the benefit that the appearance of resistance is a less common event than in the use of conventional antibiotics [16,36].

Furthermore, they also play a relevant feature in the control of viral infections of Dengue virus [37], human immunodeficiency virus [38], severe fever with thrombocytopenia syndrome virus [39], hepatitis B virus and influenza virus [40,41,42], through inhibitory mechanisms of interaction, binding and replication of the virus in the host cells.

Therefore, several works already indicated that the consumption of phenolic compounds through the daily diet offers a wide range of benefits. Even so, it is believed that some of them are still to be discovered, and in this aspect, bioinformatics tools, as the use of molecular docking that allows extensive potentials studies using the information indexed in databases, such as Phenol-Explorer (http://www.phenol-explorer.eu, accessed on 10 June 2021) or the USDA Nutrient Data Laboratory Flavonoid Database (https://www.ars.usda.gov/northeast-area/beltsville-md-bhnrc/beltsville-human-nutrition-research-center/methods-and-application-of-food-composition-laboratory/mafcl-site-pages/database-resources/, accessed on 10 June 2021), play an important role. In fact, these tools are considered effective for the search for new treatments against various diseases, once they permit modulating the molecular dynamics of these compounds with different target proteins [10,13,43,44,45,46,47].

Taking into account the described above and knowing that, generally, obese and/or diabetic individuals and people who suffer some morbidities are more susceptible to develop the most serious symptoms of COVID-19, it is not surprising that phenolics have really some anti-SARS-CoV-2 actions, as already documented by these modern tools.

### Phenolic Compounds in COVID-19

In accordance with the mentioned above, namely in the ability of phenolics to interact in viruses, multiple studies revealed phenolics can also inhibit infection by previous coronaviruses, which are, the SARS-CoV and Middle East respiratory syndrome coronavirus (MERS-CoV). Both viruses present an identic mechanism of infection when compared to that of SARS-CoV-2 [48,49,50].

Focusing on the SARS-CoV-2, its infection and continuous replication into cells can cause an excessive and non-specific immune response in some patients, originating an exacerbated production of pro-inflammatory markers (e.g., interleukins (IL), namely the IL-6, tumor necrosis factor α, macrophage inflammatory protein 1a, monocyte chemoattractant protein 1, and interferon-gamma inducible protein-10) [51,52]. Consequently, severe attacks can occur in many organs, including lungs and kidneys, leading to eventual cell death, sepsis, organs failure, and sometimes, patient death [52]. In this context, some phenolic compounds have been shown to have a determining effect on the modulation of interleukin synthesis induction routes, allowing them to be promising tools in the management of this response [53]. These can act at different levels, modulating gene expression and inhibiting the activity of certain receptors related to the initiation of chronic inflammatory response cascades, such as nuclear factor kappa-B, or mitogen-activated protein kinase and cyclooxygenase-2 [54].

**Figure 2:**
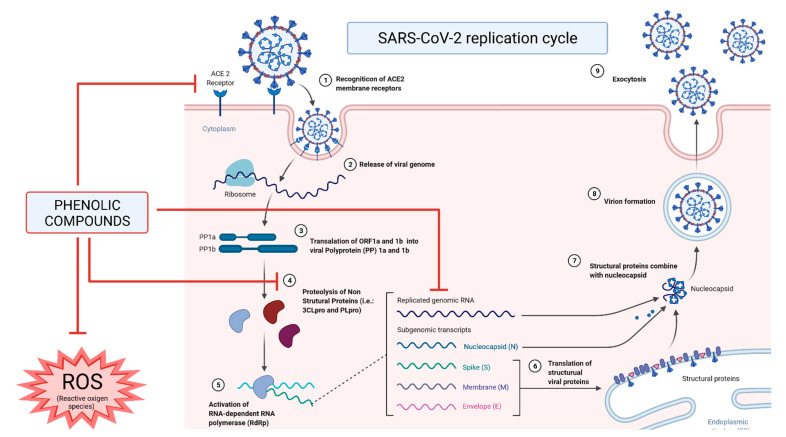
Mechanisms of action and inhibition of phenolic compounds against severe acute respiratory syndrome of coronavirus 2 (SARS-CoV-2) on its replication cycle and in the host’s immune response.

In addition to their anti-inflammatory properties, phenolics already proved to be able to prevent the entry or fusion of this virus in cells by binding to the S protein, modifying its binding site, and thus, avoiding the recognition process with the host, as described in Figure 2 [44,55,56]. Additionally, phenolics can also interact with membrane-binding receptors, mainly ACE2 proteins, which are the main point of recognition and entry into the host cell by the SARS-CoV-2 virus and block them [8]. Among phenolics, hesperetin shows a notable ability to binds with ACE2 protein, preventing the ligation of the virus S protein [44].

Phenolics can also interact with the amino acids present in the active sites of M^pro^, 3CL^pro^ and PL^pro^ proteases, interfering with their activity and blocking the synthesis of several proteins necessary for the correct replication of the virus [49,57]. Similarly, it has been observed that phenolics can also stop the activity of RdRp in an analogous way exerted by the antiviral remdesivir, and hence, prevent the replication of the virus [58,59,60].

Table 1 summarizes the main anti-SARS-CoV-2 effects attributed to phenolic compounds. 

Another aspect by which phenolic compounds can act against SARS-CoV-2 infection is due to their ability to control and manage the oxidative stress state once patients with COVID-19 presented higher total oxidative and reduced glutathione levels [61]. For this reason, it is not surprising that the daily intake of phenolics can, through a multifactorial approach at several metabolic and regulatory levels, mitigate this situation of oxidative stress and attenuate the severity of the symptoms [62,63].

**Table 1:** Anti-SARS-CoV-2 effects attributed to phenolic compounds.

Phenolic Compounds	Main Outcome	Reference
Cyanidin	M^pro^ inhibitor	[64,65,66,67]
Daidzein		
Dieckol		
Genistein		
Mearnsitrin		
Myricitrin		
Psoralidin		
Quercetin 3-*O*-β-D-glucoside		
Rutin		
Xanthoangelol E		
Benzoic acid	RdRp inhibitor	[68,69]
Cyanidin		
Daidzein		
Ellagic acid		
Gallic acid		
Genistein		
Kaempferol 3-*O*-rutinoside		
Naringenin		
Oleuropein		
Quercetin		
Quercetin 3-*O-*rutinoside		
Resveratrol		
Myrcetin	Non-structural SARS-CoV-2 helicases inhibitor	[70]
Scutellarein		
Cyanidin 3-*O*-glucoside	PL^pro^ inhibitor	[57,71,72]
Epigallocatechin		
Epigallocatechin gallate		
Hypericin		
Kaempferol		
Quercetin		
Cryptotanshinone	TMPRSS2 inhibitor	[10,70,73]
Ellagic acid		
Gallic acid		
Kaempferol		
Luteolin		
Quercetin		
Afzelin	ACE2 inhibitor	[44,70,73,74,75]
Apigenin		
Baicalin		
Biorobin		
Caffeic acid		
Catechin		
Chlorogenic acid		
Chrysin		
Ellagic acid		
Curcumin		
Cyanidin		
Delphinidin		
Epigallocatechin		
Epigallocatechin gallate		
Ferulic acid		
Galangin		
Gallic acid		
Hesperetin		
Isoferulic acid		
Kaempferol		
Luteolin		
Myricitrin		
Naringenin		
Nobiletin		
Nympholide A		
Pinocembrin		
Quercetin		
Rhoifolin		
Rutin		
Scutellarein		
Taiwanhomoflavone A		
Tangeretin		
ε-Viniferin		
Chrysin	Interact with Spike protein	[44,70,73,74,75]
Ellagic acid		
Gallic acid		
Hesperetin		
Pinocembrin		
Artepillin C	Inhibit p21-activated kinase 1	[76]
Ellagic acid	Inhibit furin	[77]
Gallic acid		

Abbreviations: Mpro, Main Protease; PL^pro^, papain-like protease; RdRp, RNA-dependent RNA polymerase; TMPRSS2, transmembrane protease serine 2; ACE2, Angiotensin-Converting Enzyme II.

## 3. Uptake of Phenolic Compounds in Dietary Supplements in COVID-19

Certain beverages have been commonly associated with high content of phenolic compounds, such as red wine, tea and coffee. For example, the consumption of red wine in moderate amounts daily, commonly a glass (~200 mL), in the Mediterranean diet seems to be associated with a cardioprotective effect, due to the presence of resveratrol [78]. Regarding SARS-CoV-2, in vitro tests applying a concentration of resveratrol between 1.56 and 200 µM in Vero cells cultures showed that this compound has a half-maximal effective concentration (IC_50_) of 4.48 µM. Additionally, the co-incubation of cells with the SARS-CoV-2 and a concentration of 50 µM of resveratrol for one hour showed to cause a reduction by 64% in virus infection, indicating a possible blockage of the virus’s ability to enter host cells [79]. In accordance with that, it has also been reported that the daily intake of 3 doses of 5.6 mg of resveratrol by COVID-19 patients showed a 50% reduction in the percentage of deaths in the treated group [80].

Furthermore, it has also been observed, under in vitro conditions, that epigallocatechin-3-gallate extracted from green tea prevents the replication of SARS coronaviruses-CoV-2, HCoV-OC43 and HCoV-229E, by inhibiting the 3CL^pro^ activity [81,82]. Moreover, other studies based on molecular docking revealed that this compound also presents a high degree of affinity with the active sites and binding sites of the S protein, PL^pro^, RdRp and ACE2 of the host, possessing binding energy equal or greater than that obtained between the host´s protein and antivirals remdesivir and favipiravir [83]. In this context, other compounds of green and black teas, particularly theaflavins (Table 2) [83], also revealed potential, through docking studies, to stop the activity of other enzymes involved in the transcription of the virus, namely of 3CL^pro^, S protein, PL^pro^ and RdRp [60,84]. These activities are not surprising and are in accordance with other studies which showed that theaflavins can, effectively, inhibit in vitro the activity of 3CL^pro^ by up to 20% at concentrations of 40 µg/mL, displaying an IC_50_ of 8.44 µg/mL [82]. Besides, theaflavins also have better binding indices compared to non-structural protein 16 (Nsp16) of SARS-CoV-2 [85].

Several flavonoids, hydroxybenzoic and hydroxycinnamic acids, and *N*-phenylpropenoyl-L-amido acids of cocoa (*Theobroma cacao*) also revealed capacity to interfere with M^pro^ activity, namely naringin, amentoflavone, nicotiflorin, isorhoifolin and rutin (Table 2) [86].

Besides, phenolics of black garlic also revealed anti-SARS-CoV-2 actions, which is very beneficial given the consumption of this foodstuff derived from the transformation of fresh garlic subjected to high temperatures (>70 °C), has become popular due to its notable antioxidant properties [87]. In this order of ideas, Nguyen et al. [88] analyzed the capacity of 49 polyphenols present in pure black garlic extract (Table 2) to inhibit the protease activity of the M^pro^ enzyme. The obtained data revealed that this extract can inhibit completely the action of M^pro^ of SARS-CoV-2, presenting an IC_50_ value of 137 ± 10 µg/mL. Among compounds, myrcetin (flavonol), naringenin (flavonone) and epigallocatechin (flavan-3-ol), vitexin (flavone) and diarylheptanoid dimethylcurcumin (curcuminoid) were the most active. Focusing on naringenin, the obtained result agrees with other in vitro studies that reported that, in fact, this compound can interact with M^pro^, showing an inhibitory IC_50_ value of 92 nM [89]. This compound is very abundant in orange, grapefruits and derivative juices, which in turn, present considerable amounts of vitamins C and D, and whose abilities to reduce the severity of symptoms caused by flu states are known [62,90,91]. However, naringenin does not seem to have a special affinity for the active site of SARS-CoV-2 RdRp, being surpassed by other molecules, such as quercetin [92].

In addition, it has also been evaluated the inhibitory capacity of a mixture of polyphenolics in different concentrations of tannic acid with puerarin, daidzein, and/or myricetin against this protease. It was reported that the most effective combination was a mix of tannic acid (5 µM), puerarin (20 µM), daidzein (20 µM) and myricetin (20 µM), which demonstrated ability to reduce M^pro^ action in the order of 80% [88].

Additionally, it has also been reported that the consumption of beverages with a high content of phenolic compounds, like coffee, can be an excellent tool in the prevention and management of this infection. These evidences are not surprising since it is well-known that the consumption of 3 and 4 cups per day of coffee exert positive effects on neurological and immune systems [93]. Therefore, the effects of 28 derivatives of caffeic acid on SARS-CoV-2 were already evaluated by docking (Table 2). All of them exhibited better docking scores than the antiviral nelfinavir, highlighting khainaoside C (−191.599 expressed in docking score against M^pro^), 6-*O*-caffeoylarbutin (−171.541 expressed in docking score against Nsp15), khainaoside B (−150.44 expressed in docking score against fusion protein S2 subunit), khainaoside C (−166.448 expressed in docking score against S protein open state) and vitexfolin A (−158.443 expressed in docking score against S protein closed state) [94]. Moreover, other cinnamic acids, namely caffeic acid and ferulic acid, also revealed ability to link with the membrane protein of SARS-CoV-2, showing binding energies of −8.4 and −8.3 kcal/mol, respectively [95].

Berries, whose consumption is increasing worldwide, due to their organoleptic characteristics and their recognized antioxidant properties, given the presence of anthocyanins, also showed capacity to interact with SARS-CoV-2 [96]. Through molecular docking analysis, among anthocyanins, cyanidin 3-arabinoside is the one that presents a more stable bond with the residues of the binding site of Mᵖʳᵒ. On the other hand, pelargonidin 3-glucoside, pelargonidin 3-rhamnoside, and cyanidin 7-arabinoside reveal ability to inhibit the linkage of SARS-CoV-2 to host cell ACE2 receptors, due to their capacity to interact competitively with S protein binding receptors [97]. Furthermore, and although cyanidin 3-*O*-glucoside shows discrete values when compared to the broad spectrum antiviral GC376, it has also been reported this cyanidin derivative shows capacity to inhibit the action of 3CL^pro^, revealing an IC_50_ value of 65.1 ± 14.6 µM [57]. Additionally, it is also important to take into account that berries possess other phenolic compounds with remarkable potential, such as quercetin, luteolin, and isorhamnetin, whose abilities to downregulate of PI3Kg-mediated PI3K-Akt signaling pathway, control pre-inflammatory cytokines production, inhibit cell proliferation, and induce apoptosis and autophagy, are also well-described [68].

Besides, golden spice turmeric (*Curcuma longa*), which is a classic ingredient of Asian food that has become popular in Western societies, due to its immunomodulatory, antioxidant and anticancer capacities, also seems to have anti-SARS-CoV-2 effects. This fact is not surprising, and it is mainly related to the presence of curcumin, as already described by several in vitro and clinical studies [98,99,100]. The administration of 140 mg of nano-curcumin showed having a modulating effect in COVID-19 patients (Table 2) by suppressing the expression of genes involved in the pro-inflammatory cytokines IL-1*β* and IL-6, IL-18, and TNF-α production [99]. That way, the cytokine storm, which is a determining factor in the severity of this disease and can lead to the most critical cases, is avoided [51,53]. Another study also observed that the percentage of deceased patients in the control group was lower (40%, 8 out of 20) compared to the group treated with nano-curcumin (20%, 4 out of 20). The treatment with nano-curcumin has also been shown to be capable of increasing the count of Treg cells, anti-inflammatory cytokines IL-10 and IL-35, and transforming growth factor (TGF)-*β* in bloodstream, as well as the expression of their related genes, being another evidence regarding the usefulness of this treatment in regulating the immune response and the severity of the disease [99,100].

The next table summarizes the main phenolic sources with potential to inhibit SARS-CoV-2 infection.

**Table 2:** Examples of the use of rich-phenolic foods and dietary supplements in SARS-CoV-2 inhibition.

Origin	Compound	Type of Study	Drug Target	References
*Theobroma cacao* (Cocoa)	Flavonoids, hydroxybenzoic acids, hydroxycinnamic acids and *N*-phenylpropenoyl-L-amido acids	Molecular docking	M^pro^	[86]
*Camellia sinensis* (green tea)	Epigallocatechin-3-gallate, theaflavin	*in vitro*	M^pro^	[81,82]
*Camellia sinensis* (green tea)	Epigallocatechin-3-gallate, theaflavin	Molecular docking	3CL^pro^, Spike protein, PL^pro^, RdRp and ACE2	[84]
*Camellia sinensis* (green tea)	16 phenolic compounds	Molecular docking	Nsp 6	[85]
Different sources	100 phenolic compounds (epigallocatechin, hesperidin, myricetin, quercetagetin and theaflavins	Molecular docking	RdRp	[60]
Berries	18 anthocyanins	Molecular docking	Spike protein and M^pro^	[97]
*Allium sativum* (black garlic)	Total extract, 49 polyphenols and 1 combination treatment of tannic acid with myricetin, puerarin, and/or daidzein	*in vitro*	M^pro^	[88]
*Euphorbia cuneata*	Naringenin	Molecular docking and *in vitro*	M^pro^	[89]
Red wine, Chinese hawthorn and blackberry	Quercetin, luteolin, and isorhamnetin	Molecular docking and metadata analysis	Several mechanisms	[68]
*Citrus*	Naringenin Neohesperidin Nobiletin	Molecular docking	Spike protein and ACE-inhibitor	[101]
*Citrus aurantium* Citri Reticulate Pericarpium	Hesperitin	Molecular docking	Spike protein and ACE-inhibitor	[75,102]
Coffee	28 caffeic acid derivatives	Molecular docking	M^pro^, Spike protein, Nsp15 and fusion protein subunit S2	[94]
Different sources	Cyanidin 3-*O*-glucoside and resveratrol	Molecular docking and *in vitro*	M^pro^	[57]
Different sources	Gallic acid, quercetin, caffeine, ribavirin, resveratrol, naringenin, benzoic acid, oleuropein, ellagic acid	Molecular docking	RdRp	[92]
*Erigenon breviscapus (Vant.)*	Scutellarin	Molecular docking	Spike protein and ACE-inhibitor	[75]
Propolis from *Apis mellifera*	Propolis (caffeic acid, p-coumaric acid, ferulic acid, *t*-cinnamic acid, hesperetin, chrysin, pinocembrin, CAPE)	Molecular docking and *in vitro*	ACE2 and Spike protein	[44]
Propolis from *Apis mellifera*	Propolis (Caffeic acid, p-coumaric acid, *t*-cinnamic acid, hesperetin, chrysin, pinocembrin, CAPE, rutin, myricetin, luteolin, resveratrol	Molecular docking	ACE2	[103]
Propolis form *Apis mellifera*	Propolis extract	Clinical trial	n.a.	[104]
*Rheum officinale* (rhubarb) *Reymoutria multiflora* tuber	Emodin	*in vitro* and in vivo	Spike protein and ACE-inhibitor	[105]
*Curcuma longa*	Nano-curcumin (micromicelar curcumin)	Clinical trials	Inmune response (Interleucine modulation)	[99]
*Moringa oliefera*	Apigenin, chlorogenic acid, chrysin, ellagic acid, myricetin, and quercetin	Molecular docking	Nsp9 and Nsp10	[106]
*Ginkgo biloba*	Amentoflavone, ginkgetin, bilobetin, isoginkgetin, sciadopitysin, kaempferol, quercetin, apigenin, isorhamnetin, genkwanin, luteolin, quercetin)	Molecular docking	M^pro^	[107]
*Glycine max* (Soya bean)	Nicotinamine	*in vitro* and in vivo	Spike protein and ACE-inhibitor	[108]
*Glycyrrhiza radix* (Licorice root)	Glycyrrhizin	*in vitro*	Spike protein and ACE-inhibitor	[75]
*Scutellaria baicalensis*	Baicalin	*in vitro*	Spike protein and ACE-inhibitor	[75]
Traditional Indian medicine plants (*Azadirachta indica, Syzygium aromaticum, Ocimum sanctum, Zingiber officinale, Curcuma longa, Camellia sinensis, Nigella sativa, Luffa cylindrica, Allium sativum and Allium sativum*)	Nimbaflavone, epigallocatechin, catechins and curcumin	Molecular docking	M^pro^	[109]
Several plants from traditional Indian medicine	23 bioactive compounds, including quercetin, apigenin, curcumin, carvacrol and gingerol	Molecular docking	ACE2, M^pro^, Spike protein, RdRp	[66]
Traditional Chinese medicine formulations	Kaempferol, luteolin, isorhamnetin, epigallocatechin-3-gallate, naringenin and wogonin	Molecular docking, in vitro and clinical trials	M^pro^	[110]
Traditional Chinese medicine formulations	Isorhamnetin	Molecular docking, in vitro and clinical trials	ACE2 and M^pro^	[111]

Abbreviations: Mpro, Main Protease; PL^pro^, papain-like protease; RdRp: RNA-dependent RNA polymerase TMPRSS2: transmembrane protease serine 2; ACE2: Angiotensin-Converting Enzyme II; Nsp: Non-structural protein; n.a: not applicable.

Although docking studies are an asset, more studies, namely clinical trials, are necessary to unravel the full potential and safe dosage of these food sources, and related phenolics, in preventing SARS-CoV-2 infection.

## 4. Uptake of Phenolic Compounds in Dietary Supplements in COVID-19

Currently, there is a wide availability of dietary supplements based on foods and plants with nutraceutical value, in order to improve the supply of nutrients or molecules in which the diet could be deficient [112,113]. This fact shows an upward trend due to the ease of consuming concentrated formulations derived from foods or natural products [114]. Generally, phenolic compounds and plants where they are found spontaneously are one of the most attractive ingredients for this type of preparation, since their consumption is not always carried out and thus, their use facilitates their incorporation into the diet [115,116]. Besides, it is also correct to include here traditional medicines, such as the Chinese and Indian ones, since both include plants rich in several bioactive compounds, like garlic or onion and other spices rich in antioxidant compounds, useful to treat different conditions [68,117,118,119,120,121].

One of the most popular plants commonly used as a dietary supplement, *Moringa oleifera*, also called “miracle tree” owing to its distinguished anti-inflammatory, anti-cancer and anti-diabetic properties [122]. This plant is very rich in several phenolic compounds, standing out apigenin, chlorogenic acid, chrysin, ellagic acid, myricetin and quercetin (Table 2), whose binding capacity with the Nsp9 and Nsp10 of COVID-19 was already described. Between them, ellagic acid was the one that presented lower binding energy compared to Nsp9 (−6.9 kcal/mol) and apigenin, the one that presented the lower binding energy compared to nsp10 (−7.1 kcal/mol). Both compounds exhibit a constant inhibition (Ki) of 5.98 µM [106]. Another plant largely used due to its antioxidant qualities, and cardioprotective and neuroprotective effects is *Ginkgo biloba* (Table 2). This one is composed of numerous phenolics, such as flavones (amentoflavone, ginkgetin, bilobetin, isoginkgetin, sciadopitysin, apigenin and luteolin) and already revealed, by molecular docking, ability to inhibit the 3CL^pro^ of SARS-CoV-2 (IC_50_ value < 10 µM) [107,123].

Besides, the consumption of phenolic compounds can also occur by ingestion of products derived from plants but obtained by animal activity, such as propolis. Propolis is produced by bees and shows important antibiotic and anticoagulant activities, being considered traditional medicine in several cultures. Given the mentioned, it is currently sold in the form of hydroethanolic extract throughout the world [103]. Recent in silico data revealed that propolis extracts rich in phenolics can inhibit the binding of the S protein S1 of the SARS-CoV-2 virus with the ACE2 proteins [44]. Among phenolics, hesperetin was the one that presented a lower IC_50_ value (16.88 mM), followed by pinocembrim (29.53 mM) and CAPE (79.09 mM) [103]. Furthermore, hesperetin also proved to have ability to interact with 3CL^pro^ of SARS-CoV [44,49,124]. Concerning clinical assays, the intake of propolis extracts supplementation at different oral doses (400 mg/day and 800 mg/day) during 7 days in hospitalized COVID-19 patients, revealed a positive effect on the reduction in their average hospital stay of patients, reducing from 12 days in the control group to 7 days for those treated with the dose of 400 mg/day and to 6 days for those treated with 800 mg/day (Table 2). In addition, another significant aspect was the reduction in the number of patients who developed kidney injury in the treatment with the highest dose (800 mg/day), where it was observed that 4.8% of the patients had acute kidney injury in the 800 mg/day group, 12.5% in the 400 mg/day group and 23.8% in the control group confirming a correlation between propolis administration and amelioration of kidney damage [104].

Focusing on traditional Chinese medicine, this one presents a wide number of supplements and formulations based on plants and extracts that have shown to have significant potential in the treatment of different respiratory diseases, including against COVID-19. A study based on the treatment of COVID-19 patients by the consumption of nine different Chinese formulations revealed that three of them (Table 2) exerted positive effects against the virus. Additionally, an in silico analysis indicated that these benefits are essentially due to quercetin, kaempferol, luteolin, isorhamnetin, epigallocatechin-3-gallate, naringenin, and wogonin (Table 2). Between these compounds, epigallocatechin-3-gallate was the one with the lowest binding energy (−7.9 kcal/mol), revealing an IC_50_ score for 3CL^pro^ (IC_50_ = 0.67 ± 0.09 µM) [110]. To complete the previous study, another metadata analysis of 16 Chinese formulations showed that isorhamnetin has also ability to bind with the ACE2 and 3CL^pro^, regulating inflammation, cellular processes, and endocrine system [111].

Traditional Indian medicine, or Ayurvedic medicine, is also a source of important bioactive compounds, in this case through the use of mixtures of plants, some of them considered spices and food plants such as garlic or pepper [125]. Using molecular docking, the activity against SARS-CoV-2 of 15 compounds present in plants used in traditional Indian medicine, as *Azadirachta indica*, *Syzygium aromaticum*, *Ocimum sanctum*, *Zingiber officinale*, *Curcuma longa*, *Camellia sinensis*, *Nigella sativa*, *Luffa cylindrica* and *Allium sativum* has been evaluated. The obtained results indicated that nimbaflavone, epigallocatechin, catechins and curcumin (Table 2) are the main responsible for the anti-SARS-CoV-2 activities, standing out the effects of nimbaflavone, which shows lower binding energy (−8 kcal/mol) and a Ki of 1370.95 nM [109]. Another study also revealed the notable action of curcumin against this virus, particularly due to a higher binding affinity with the ACE2 protein receptor (−88.9, expressed as docking score), and also with the receptor-binding domain of the Spike protein (−109.8), 3CL^pro^ (−115.69) and RdRp (−97) [66]. Moreover, the supplementation with four different formulations of Ayurvedic medicine in doses of 1 g/day divided into 2 daily capsules showed a greater and faster recovery, with a complete recovery of 100% of the patients on day 21, as opposed to the control group, where only 70% had recovered at the same time [125].

## 5. Population Experiences of Phenolic Compounds, Diet and COVID-19

A remarkable aspect of today’s society is the awareness that citizens present of the influence that diet has on their general state of health, considering it an added value to achieve a full state of health [126]. It is for this reason that emerged the concept of functional food, where one molecule/compound, which is part of a regular diet is capable to provide a benefit to the health of individuals without the need to be supplemented [127]. This concept was already shared by Hippocrates, who considered that food should be the best medicine [128]. Thus, a special interest has been revealed during the times of the pandemic, in which Internet searches for information about food or supplementary plant-based treatments have increased in a similar way to the search for tags such as “coronavirus” or “COVID” [126]. This fact is due to the importance of having a competent immune system capable to modulate the COVID-19 infection and the response to it, which can be achieved through a complete and balanced diet, and the intake of dietary supplements [63]. For this reason, interest in the development of supplementation therapies based on the active properties of different foods to increase the contribution of active compounds able to act as a preventive approach against viral diseases [112].

Focusing on COVID-19, it was already verified that the ingestion of supplements based on *Zingiber officinale*, *Curcuma longa*, vitamins and other micronutrients can be a complementary treatment in COVID-19 patients, given their ability to boost the immune system [129]. Particularly, there has already been demonstrated that the intake of vitamins C and D can reduce mortality and modulate cytokine storm associated with the most severe symptoms of COVID-19 [90,130]. Besides, positive correlations regarding the regular consumption of fish and mollusks, meat, eggs milk and vegetables, and better recovery of infected patients have also been reported worldwide [131]. In addition, there is evidence of the consumption of phenolic-rich fruits and vegetables can also meliorate respiratory infections [130].

Among diets, recent evidence revealed the Mediterranean one can have a notable capacity in attenuating symptoms caused by SARS-CoV-2, by improving antioxidant capacities, modulating pre-inflammatory immune parameters and reducing the cardiovascular risk [78,132,133]. A comparative study between Spain, a country associated with a Mediterranean diet, and other countries with less adherence to this diet, revealed that the continuous ingestion of foods found in Mediterranean diet can protect people from damage caused by SARS-CoV-2, inclusive individuals who are more susceptible to develop serious symptoms of COVID-19 (e.g., obese, diabetic, among others) [78,133]. On the other hand, the adherence to a Western diet, which is rich in fats, can increase the severity of this infection due to the raise of pre-inflammatory cytokines, free radicals and reactive species levels in plasma, and the risk of hyperglycemia and hyperlipidemia states [51].

Besides the mentioned, nowadays it is increasingly accepted that metadata analysis can help to determine the influence of certain foods in some diseases, including COVID-19. For example, Xu et al. [68] analyzed, by in silico, about 12,000 phenolic compounds and their capacity to inhibit the SARS-CoV-2 3CL^pro^, and verified that the consumption of red wine, Chinese hawthorn, and blackberry is a source of polyphenols very useful to combat this virus. Without a doubt that this analysis may be a new approach to determine new nutritional strategies to conduct clinical trials in order to determine the nutraceutical properties of foods.

## 6. Conclusions and Future Remarks

Disponible studies show that phenolic compounds present in most food and dietary supplements are capable to inhibit the SARS-CoV-2 virus, namely through competitive linkage to the active sites of the different proteases or polymerases of the virus, and hence complicating the entrance of the virus into cells. Among them, curcumin, epigallocatechin-3-gallate, resveratrol and theaflavin have been shown to be effective in impeding the virus using in silico and in vitro tools. Resveratrol also showed preeminent results in clinical trials. Besides, herbal formulations rich in phenolic compounds and the supplementation with propolis seem to have a prominent effect in infected COVID-19 patients, improving recovery and reducing mortality. Although these data are promising, it is important to know that many of the available trials have been carried out at a theoretical level, using bioinformatics simulation techniques. Given that, it is essential to carry out more clinical trials to assess the full biological potential of these compounds and unravel their safe and adequate dosage, even because, some phenolics present low solubility and poor bioavailability, and when they are consumed at high doses, they can act as pro-oxidants. Even so, population-level studies show that diets rich in phenolic compounds, such as the Mediterranean diet, can lower the incidence of many diseases and exhibit therapeutic effects, including on COVID-19. For this reason, the adopted strategies to attenuate, or even mitigate, this pandemic disease should also consider a nutraceutical approach. Among the alternatives, the best one is the adoption of a daily diet rich in natural antioxidant compounds, which already showed to be useful to alleviate the symptoms and the incidence of SARS-CoV-2. Additionally, another useful tool is their incorporation in dietary supplements, pharmaceuticals and nutraceuticals.
